# Do people really care less about their cats than about their dogs? A comparative study in three European countries

**DOI:** 10.3389/fvets.2023.1237547

**Published:** 2023-10-23

**Authors:** Peter Sandøe, Clare Palmer, Sandra A. Corr, Svenja Springer, Thomas Bøker Lund

**Affiliations:** ^1^Department of Food and Resource Economics, University of Copenhagen, Frederiksberg, Denmark; ^2^Department of Veterinary and Animal Sciences, University of Copenhagen, Frederiksberg, Denmark; ^3^Department of Philosophy, Texas A&M University, College Station, TX, United States; ^4^Division of Small Animal Clinical Sciences, School of Biodiversity, One Health and Veterinary Medicine, University of Glasgow, Glasgow, United Kingdom; ^5^Unit of Ethics and Human-Animal Studies, Messerli Research Institute, University of Veterinary Medicine, Vienna, Medical University of Vienna, University of Vienna, Vienna, Austria

**Keywords:** companion animals, attachment, dogs (canis familiaris), cats (felis catus), LAPS, pet health insurance, willingness to pay, representative questionnaire-based survey

## Abstract

Previous studies have shown that cat owners seem to care less about their cats than dog owners care about their dogs – both in terms of their emotional state of attachment and in their willingness to pay for services that potentially benefit the animals. One study speculated that this difference is “driven by the behavior of the pet” – that the behavior of dogs encourages care more than the behavior of cats – and therefore is a universal phenomenon. However, previous studies mostly relied on convenience sampling of owners and were undertaken in single countries. Based on responses to a questionnaire from cat and dog owners drawn from representative samples of citizens (18 to 89 years of age) in three different European countries, Denmark, Austria and the United Kingdom, we tested the degree to which owners care about their cats and dogs. We used four different measures: Lexington attachment to pets scale (LAPS), possession of pet health insurance, willingness to pay for life-saving treatment, and expectation of veterinary diagnostic and treatment options. Dog owners had higher LAPS scores in all countries. However, the difference between dog and cat owners was greater in Denmark than in Austria and the United Kingdom. More dogs than cats were insured in all three countries, but the ratio was much less skewed in favor of dogs in the United Kingdom compared to Denmark. In terms of expensive life-saving treatment, in every country, more dog owners than cat owners were willing to spend over a certain amount, but the differences were much more pronounced in Denmark compared to the United Kingdom. In Denmark and Austria, dog owners expected more veterinary treatment options to be available, but species made no difference to the expectations of UK owners. People care more about their dogs than their cats in all countries, but with a clear cross-country variation and a very modest difference in the United Kingdom. Therefore, it does not seem to be a universal phenomenon that people care much less about their cats than their dogs. This finding has practical implications for future efforts to expand the level of veterinary services provided for cat owners.

## 1. Background

The present study compares the degree to which dog owners are attached to or otherwise care about their dogs with how much cat owners are attached to or otherwise care about their cats. In line with the literature, we will take a broad view of what it means to care about a pet. We will include both an emotional state of attachment and a willingness to pay for services that potentially benefit the pet. In line with much literature in economics, we see a link between the two, where the sincerity of peoples’ emotional claims should be reflected by a willingness to “put their money where their mouth is.”

Two existing studies (1, 2) used the Lexington Attachment to Pets Scale (LAPS) which asks owners to respond to a series of 23 statements such as ‘I believe that my pet is my best friend’ (2). One of these studies (2) found a higher LAPS score among those pet owners who identified dogs rather than cats as their favorite pet, and the other (1) found that dog owners in general scored higher than cat owners.

Other studies have used the Family Bondedness Scale (3), the MDORS scale for dogs and the CORS for cats (4), the Owner-Pet Relationship Scale (5) and the Comfort from Companion Animal Scale (6). In general, these studies also found that dog owners scored more highly than cat owners. However, of the two most recent studies, one (3) only found an “extremely small effect size” (p. 6), and the other (4) found mixed results.

Difference in the willingness to pay for and/or provide a certain level of health care for the animal is another commonly used measure to compare the degree to which people care about dogs and cats. One study (7) measured willingness to seek out veterinary care for dogs and cats, finding that cats are seen by veterinarians significantly less often than dogs, even in households that contain both species. Lue et al. (7) also measured the amounts that dog and cat owners were willing to spend on life-saving treatment for their animals and found that dog owners were willing to spend significantly more. Freiwald et al. (8) found that a greater number of dog vs. cat owners were willing to spend $1,000 or more on dealing with various health problems. Finally, Kirk (9) looked at willingness to pay for life-saving treatment as a measure of how much owners care about their dogs and cats, but in addition considered possession of health insurance, finding that more people insured their dogs than their cats.

Other measures such as the use of veterinary services and time spent with the pet, show the same pattern, namely, that people care more about their dogs than about their cats. Thus, Kirk (9) found owners were more willing to buy items for their dogs than their cats. Lue et al. (7) found dogs were more frequently taken to the vet, vaccinated and given preventative dental care, and that in general more time was spent with a dog than with a cat, that the dog was more likely to be considered as a child, that a dog was more missed when the owner was away from home, and that owners were more likely to buy gifts for their dogs than for their cats. In addition, Nielsen et al. (10) found that dogs were much more likely to be vaccinated according to recommendations than cats. Finally, Freiwald et al. (8) found that dog owners were more likely to vaccinate their pet and to give pills twice a day for 1 month than cat owners, and that dog owners were more likely than cat owners to spend 10 minutes three times a day for 6 weeks to train their pet.

Various explanations have been offered for these differences in level of attachment or willingness to pay for various services relating to the pet. One explanation is that people do more with their dogs than their cats, for example, taking dogs for walks and training them (5). Lue et al. (7) found that owners of both dogs and cats thought that dogs were more affectionate and just more fun to be with than cats. A further study based on the Monash dog owner relationship scale found that even though owners in households with both cats and dogs had a better relationship with their cats because they found cats easier to look after, they felt emotionally closer to their dogs (4). Some studies suggest that humans are responding to perceptions of the animals’ own differing attachments to humans, in particular that cats appear more independent and less attached to their owners, and therefore are less often regarded as family members than dogs (11). In line with this, Potter and Mills (12) found that cats typically do not develop as strong an attachment to their owners as dogs. A further study argues that the stronger the owner’s perceived behavioral control over an animal, the stronger their sense of psychological ownership becomes; and that this sense of psychological ownership is what drives emotional attachment (9). Since owners perceive themselves to have more behavioral control over dogs than cats, according to this argument, they therefore care more about their dogs (9).

According to Kirk (9) the difference in levels of human care relating to members of the two species is “driven by the behavior of the pet.” This is, according to the author, underpinned by the finding that when studying dogs with cat-like behavior, or cats with dog-like behavior, the evaluation was reversed (9). One may call this the *dog* vs. *cat behavior hypothesis.* In this view, the typical disparity between how much owners care about their cats relative to how much they care about their dogs is based on a natural human response to the respective behaviors of cats and dogs. A competing hypothesis could be that the degree to which the majority of humans care about their cats and dogs, and indeed companion animals in general, is shaped by ideas, conventions, and historical contingencies, including the ways in which the animals are used and housed, that are culturally specific. This could be called the *culture hypothesis*.

A way of testing which hypothesis is most credible is to compare representative samples of groups of cat and dog owners across different countries. However, while the dog vs. cat behavior hypothesis seems to find support in the bulk of papers that have compared how much people care about cats and dogs, it must be emphasized that with the exception of two studies (2, 7), these studies have been based on convenience samples of owners and not on samples which are representative for key demographic traits of owner such as gender, age and education. Nor are they able to compare possible differences across countries, as each study focusses on a single country: Japan (11), Mexico (4), Slovenia (5), Germany (1), Scotland (13), or the United States (3, 6–9).

In the present paper we aim to evaluate the credibility of the two hypotheses proposed above by means of responses from representative samples of cat and dog owners from three different European countries. If we can find a similar pattern of owners caring more about dogs than about cats across the countries this would tend to support the dog vs. cat behavior hypothesis. If such consistency is not found, this would tend to support the culture hypothesis.

We made use of three measures of attachment or other aspects of caring about one’s pet used by the previous studies in the field: the LAPS, possession of pet health insurance and willingness to pay for life-saving treatment. Compared to previous studies, we added one additional measure: the level of owners’ expectation that different levels of veterinary equipment would be available to diagnose and treat dogs or cats in the practice/clinic owners usually attend with their animal. This novel way to address care rests on the assumption that people who expect more advanced veterinary treatment options to be available are likely to care more for their pet, and to expect alternatives to euthanasia if their dog or cat becomes seriously ill (but it may, of course, also in part be shaped by what owners are used to in light of current standards in veterinary practice).

The data used originates from a questionnaire study on expectations of veterinary care among owners of dogs and cats, collected from representative samples of the adult populations (18–89 years of age) in Austria, Denmark and the United Kingdom. These countries were chosen for the larger survey to reflect diversity in terms of how the veterinary sector has developed.

Viewed from a global perspective the three countries are very similar: They are all wealthy, and are highly urbanized. One relevant difference may however be in the distance from their agricultural past, and the onset of urbanization. Thus, the United Kingdom was largely urbanized by the end of the 16th century (14), while the majority of the Danish population worked in the agricultural sector until the end of the 19th century (15). Since Austria has only existed in its current shape since 1918, it is difficult to assess exactly when urbanization happened, but it seems to be earlier than in Denmark. We thought it possible that these historical patterns might lead to cross-country differences concerning how much owners care about their cats compared to how much they care about their dogs, because in traditional rural environments, cats were abundant and were typically not allowed indoors and were less attached to humans. They were mostly what may be called barn cats or semi-feral cats that inhabited farms relatively independently of the people living there – unlike dogs who have typically had a working relation to the farmer or other workers. Given this culturally-specific relation to cats, it would not be surprising were cats to occupy a place further down the so-called sociozoological scale (16) than dogs in countries where the agricultural past was quite recent (like Denmark) but would occupy a higher place closer to dogs in countries that were urbanized long ago (like the United Kingdom and Austria).

Keeping this in mind, in this study we compare the degree to which owners of cats and owners of dogs care about their respective pets in three European countries. Specifically, we aim to evaluate whether owners care about their dogs and cats to the same degree in all three countries, using the described measures. To ensure that the identified differences do not reflect country variation relating to other owner characteristics, we controlled for socio-demographic factors, and whether the reason for acquiring the animal(s) was for human companionship. We discuss our findings with a view to assessing whether they lend support to the dog vs. cat behavior hypothesis or whether there is a difference between the relative level of care between the countries that may support the culture hypothesis.

## 2. Materials and methods

### 2.1. Study populations and recruitment of participants

In the present paper, we made use of responses to selected parts of a questionnaire, which together with the data from the questions that are used in this paper, is available in the repository Zenodo.^1^

Participants were recruited by Norstat, a European-based survey company, with the aim of gaining a representative sample of Austrian, Danish and UK citizens, including pet owners. The survey company administers and hosts online panels comprising citizens from many European countries. We aimed for a sample that is representative in terms of age, gender, and region. Therefore, a stratified sampling principle was set up where individuals within each stratum were randomly invited to participate. The invitations were issued via an e-mail that contained a link to the online questionnaire. Data were collected from 11 to 25th of March 2022 in Austria, from 11 to 24th of March 2022 in Denmark and from 8 to 23rd of March 2022 in the United Kingdom. The invitation provided information about the background of the study, the participating universities, ethical approval, and estimated time for questionnaire completion. Participants were informed that the completion of the questionnaire was voluntary and anonymous, and that they could exit the survey at any point. Before participants were directed to the survey, they confirmed that they were over 17 years old, and that they consented to participate in this survey.

### 2.2. Study participants

In total, 17,747 citizens were invited to participate in the study: 5,207 in Austria, 6,075 in Denmark and 6,465 in the United Kingdom. Of these, 4,885 clicked on the survey link, but 275 questionnaires were excluded due to being incomplete (dropout rate 5.62%). The final study sample included 1,500 Austrians, 1,552 Danes and 1,558 UK citizens. The response rate was 30.34% for Austria, 27.49% for Denmark and 25.29% for the United Kingdom. The specific target group of the present study was dog and/or cat owners. We singled this group out by asking respondents if they had a companion animal in the household. If they answered affirmatively, they were then asked which animal species they had, and how many they had of the species. In total, 2,117 respondents reported that they were dog and/or cat owners; 844 dog owners, 872 cat owners and 401 owners who keep both dog(s) and cat(s). Country-specific sample sizes are presented in Table 1 (Sample size of different owner profiles).

**Table 1:** Overview of sample size of different owner profiles and sample sizes across three of the measures of how much people care about their dogs and cats employed in the study – divided into countries.

	Austria	Denmark	United Kingdom
Sample size of different owner profiles			
Dog owners	225	308	311
Cat owners	391	241	240
Dog and cat owners	184	77	140
Sample size of owners responding to LAPS^1^			
Dog owners	332	359	393
Cat owners	443	250	267
Sample size of owner responses to question about pet health insurance coverage^2^			
Dogs	526	457	568
Cats	921	459	560
Sample size of owner responses to question about willingness to pay for life-saving treatment^3^			
Dogs	409	385	451
Cats	575	318	380

1 Sample size is lower than the Owner profile count because respondents only responded to the LAPS items regarding the species they reported was their favorite pet.

2 Sample sizes are higher than the Owner profile count because respondents were asked about possession of health insurance for all cats/dogs in the household.

3 Sample sizes are higher than the Owner profile count because respondents that had both cats and dogs were asked about willingness to pay for each species.

The sample size for the fourth measure, expectation of availability of veterinary treatment, is identical to the sample size of different owner profiles.

### 2.3. Questionnaire content and measures

The first measure we used to assess how much people care about their dogs and cats was the *LAPS.* As in (2), respondents were asked which species their favorite pet was, and then instructed to think about that favorite pet when responding to the 23 LAPS statements. Among respondents that mentioned a cat or a dog as their favorite pet, the measure exhibited very good internal consistency in all three countries (Cronbach’s α: Austria_LAPS_ = 0.94; Denmark_LAPS_ = 0.95; the UK_LAPS_ = 0.94). We followed the standard procedure for construction of the LAPS which gives a scale range from 0 to 69 (2). The sample size in this analysis is reported in Table 1. Since not all owners declared their dog or cat as their favorite pet, the sample size is lower than the owner profile count.

The second measure of care, *Possession of health insurance*, was recorded by asking owners whether the cat or dog was insured, up to a maximum of three dogs and/or three cats. In the few cases where a respondent had more than three of each, they were asked to report the insurance status of the three cats and dogs, respectively, whose name came first in the alphabet. In total, we registered the insurance status of 3,491 dogs and cats. See a further breakdown in Table 1 (Number of owner responses to question about pet health insurance coverage). We used this count as a way to report the proportion of cats and dogs that were insured. We also reported the number of households that have at least one insurance policy for one of their dogs, and at least one insurance policy for one of their cats. A limiting factor when using companion animal health insurance as a measure of level of care, especially across countries, is that there are different types and levels of insurance, e.g., in terms of diseases covered, or payout limits, both within and across countries (17). Still, a comparison of the relative number of dogs and cats that are insured is of value as a measure of how much owners care for their animals, as shown by Kirk (9).

Another measure of care, willingness *to pay for life-saving treatment* (WTP), is based on a question where owners were presented with an imagined scenario. The question was formulated in two ways to take into account whether or not the pets were insured. The first formulation was: “If (one of) your uninsured cat(s) [or dog(s)] were suffering from a severe illness, and would have to either undergo treatment (with a good chance of a successful outcome), or be euthanized, what would you do?” Response options to this question ranged from “I would ask for euthanasia” to spending over seven amounts [starting at “I would spend < £100 on treatment” to “I would spend £8,000 or more on treatment” (modified into country-specific currencies, i.e., EURO in Austria and DKK in Denmark)]. Further, there was an “I do not know” option. Alternatively, owners who had insured at least one cat or dog, respectively, were asked how much they would pay in the same imagined scenario (they were give the same response options but “uninsured” cat or dog was replaced with “insured”). If owners had both an insured and uninsured cat or dog, respectively, they received both questions. The sample size for this measure is laid out in Table 1 (Number of owner responses to question about willingness to pay for life-saving treatment).

The fourth measure, *Expectation of availability of veterinary treatment*, is based on responses to the question: “Which of the following diagnostic and treatment options would you expect to be available in the practice you usually attend? Please tick all that apply.” There were eight options [radiography, ultrasound, endoscopy, arthroscopy, in-house laboratory, dental equipment, MRI scanner, CT scanner (as well as “none of the above,” and “I do not know”)]. A summary score was then created, indicating the number of options ticked by the respondents giving a variable range of 0 to 8. The same set of questions were given to all respondents irrespective of owner profile. Therefore, the sample size is identical to the owner profile counts in Table 1.

Respondents’ age (in years) and gender were recorded. Further, respondents were asked about their household income level where 11 income brackets were provided as response options (with country-specific currencies) along with two opt out options (“I do not know” and “I do not wish to say”). We collapsed these responses into a variable with three values indicating high income (approximately 1/3 of respondents), not high income, and income undisclosed. We provide an overview of the socio-demographic variables in Table 2.

**Table 2:** The socio-demographic characteristics of the three samples.

	Austria (*n* = 1,500)		Denmark (*n* = 1,552)		UK (*n* = 1,558)	
Gender						
Male	641	(314)	698	(247)	696	(292)
Female	854	(482)	851	(378)	858	(396)
Neither of these	4	(4)	1	(1)	3	(3)
Prefer not to say	1	(0)	2	(0)	1	(0)
Household income						
High income^1^	535	(297)	570	(267)	586	(308)
Not high income^2^	690	(367)	709	(265)	796	(313)
Not disclosed^3^	275	(136)	273	(94)	176	(70)
Age						
18–29 years	355	(216)	303	(103)	334	(174)
30–39 years	269	(143)	220	(100)	228	(113)
40–49 years	264	(138)	264	(143)	263	(118)
50–59 years	260	(162)	261	(129)	253	(122)
60 years or more	352	(141)	504	(151)	480	(164)

Number of owners of dogs and/or cats are reported in parentheses. ^1^Annual household income. Austria = 40,350€ or above; Denmark = 500,001 DKK or more; UK = £33,600 or more.

2 Annual household income: Austria = 40,349€ or less; Denmark = 500,000 DKK or less; UK = £33,599 or less.

3 Those who responded “prefer not to answer” and “do not know”.

The reasons for acquiring a dog or cat were also recorded where one of the options was human companionship. Table 3 gives an overview of the share of dogs and cats that were acquired with human companionship in mind.

**Table 3:** Share of dogs and cats across the three countries acquired for the purpose of human companionship.^1^

	Austria		Denmark		UK	
	Dogs (*n* = 409)	Cats (*n* = 575)	Dogs (*n* = 385)	Cats (*n* = 318)	Dogs (*n* = 451)	Cats (*n* = 380)
To provide companionship (for humans)	76%	74%	82%	77%	74%	72%

1 Question formulation was “Why was/were the dog(s)/cat(s) acquired? Choose all that are relevant for you”.

### 2.4. Data analysis

First, we examined within-country differences in the level to which people care about their cats and dogs.

For LAPS, the mean score was reported for owners that chose a dog or cat, respectively, as their favorite pet. We examined whether the mean score was different using a linear regression with LAPS inserted as dependent variable, and favorite species (cat or dog) as categorical independent variable. To ensure that the attachment differences were not due to socio-demographic factors in cat and dog owner profiles nor due to the reason for acquisition, the following four control variables were included in the regression: age, gender, income, and whether animals were acquired for human companionship (all were inserted as categorical predictors). Due to very few responses on the “Neither of these” and “I do not wish to say” options on the gender identification variable these observations were excluded from the analysis of LAPS (and all other multivariate analyses described later in this section). We compared the likelihood ratio χ^2^ values (and the appropriate degrees of freedom) before and after the main effect of species (cat or dog) was entered into the regression. We concluded that there is a statistically significant difference between cats and dogs if the model with the main effect of species is significantly better than the model without species, as determined by the difference in the likelihood ratio χ^2^ test.

Next, with respect to possession of health insurance, we reported the proportions of cats and dogs that were insured, and tested for proportional differences. We further reported the proportion of households with insurance for cats and dogs, respectively. Also, we tested whether the difference in households’ possession of health insurance (0 = no insurance; 1 = insurance) for cats and dogs was statistically significant using a mixed effect logistic regression. A mixed effect model was used because the data is clustered, as respondents that owned both a cat and dog reported the insurance status for both species. Owner id-number was included as random intercept, and species (cat or dog) as categorical indicator. The socio-demographic variables and reason for acquisition mentioned above were entered as control variables.

We then examined differences in willingness to pay (WTP) for life-saving treatment. The proportion of owners that were willing to pay a large amount for life-saving treatment for their cat or dog, respectively, was reported, and we tested whether the difference in WTP (none/low WTP = 0; high WTP = 1) was statistically significant for cats and dogs using a mixed effect logistic regression. Owner id was included as random intercept, and species (cat or dog) as categorical indicator. The socio-demographic variables and reason for acquisition mentioned above were entered as control variables.

With respect to expectation of availability of veterinary treatment options, the mean number of treatment options was reported for each owner profile. We treated expectation as an ordered variable, and used ordered logistic regression to test whether there was a statistically significant difference between owner profiles (household with cat, dog, or both). Owner profile was inserted as a categorical, independent variable. The socio-demographic variables mentioned above and reason for acquisition were entered as control variables.

To study dog-cat differences between countries, the country-level data were pooled. The following six independent variables were inserted as main effects: country, age, gender, income, reason for acquisition, and species (i.e., cat or dog, and for the Expectation indicator: owner profile). Age, gender, income, and reason for acquisition were treated as control variables and possible statistically significant effects from them were not reported. To test cat-dog country differences, we inserted the interaction effect between country and species. If the model with the interaction effect was significantly better than the model where there are only main effects, as determined by the difference in the likelihood ratio χ^2^ test, we then went on to conduct pairwise country comparisons to identify the specific countries that differed. We evaluated if two countries differed based on whether the interaction effect between country and species was statistically significant. For each measure of attachment or care, regression techniques similar to those described for detection of within country-differences were used (i.e., LAPS: linear regression, insurance and WTP: mixed effect logistic regression, and Expectation: ordered logistic regression).

For all mean scores and proportions reported, the data were weighted on gender, age, and region of the country using Stata’s *svy* command. This means that the reported results were representative for the three populations regarding these three factors.

All analyses were run in Stata/MP 17.0 for Windows. We considered effects to be statistically significant if *p* < 0.05.

## 3. Results

### 3.1. Attachment of owners to their dogs and cats as measured by LAPS

In Table 4, we present the LAPS scores for owners of dogs and cats, respectively, in the three countries. In all three countries, the mean for the LAPS was highest among owners that reported a dog as their favorite pet [which includes both owners who had only one or more dogs and owners who had dog(s) and cat(s) but who stated that a dog was their favorite pet].

**Table 4:** Mean LAPS score divided into the owner-reported favorite species (cat or dog) – across countries.

Country	Dog		Cat		*P* value^1^
	Mean	(95% CI)	Mean	(95% CI)	
Austria (*n* = 775)	52.1	(50.8, 53.4)	46.9	(45.6, 48.1)	χ^2^(1) = 26.28; *p* < 0.001
Denmark (*n* = 609)	47.3	(46.0, 48.7)	38.9	(37.1, 40.7)	χ^2^(1) = 67.32; p < 0.001
UK (*n* = 660)	51.2	(50.1, 52.4)	48.5	(46.9, 50.0)	χ^2^(1) = 5.57; p < 0.05

1 Likelihood ratio χ^2^ test result from linear regression. Age, gender, income and reason for acquisition were included as control variables in the regression.

Unweighted sample sizes are reported in the table.

We found the largest relative difference in attachment between dog and cat owners in Denmark (LAPS Mean_cats_ = 38.9 compared with LAPS Mean_dogs_ = 47.3). Further tests confirmed that the cat/dog difference in attachment is higher in Denmark compared to Austria [LR χ^2^(1) = 6.82, *p* < 0.01] and the United Kingdom [LR χ^2^(1) = 12.91, *p* < 0.001]. We found that the relative difference in attachment between dog and cat owners did not differ between Austria and the United Kingdom.

### 3.2. Possession of health insurance

In all three countries, more dogs than cats were insured, and results from the tests of proportional difference were also significant in all countries (Table 5). When considered at household level, once again, dogs were more often insured than cats (Table 6). The reported proportions in Tables 5, 6 also indicate that it was much more likely that dogs than cats were insured in Denmark (48%-point difference), compared to Austria and the United Kingdom (both showing a 19%-point difference). Further tests confirmed that the difference was statistically significant when comparing household level insurance percentages in Denmark and Austria [LR χ^2^(1) = 23.06, *p* < 0.001], and Denmark and the United Kingdom [LR χ^2^(1) = 47.08, *p* < 0.001]. In the comparison between Austria and the United Kingdom, a statistically significant difference was also observed [LR χ^2^(1) = 3.97, *p* < 0.05]. This may seem counterintuitive when the %-point difference is similar. This may be due to control variables modifying the effect of species. It may also reflect that the likelihood of possessing health insurance for a dog relative to a cat is different in the two countries (2.5 in Austria, and 1.5 in the United Kingdom; calculated on basis of proportions in Table 6).

**Table 5:** Percentage of dogs and cats that are insured in each country.

	Dogs	Cats	*P* value^1^
Austria (*n* = 1,447)	29%	10%	Z = 9.53; *p* < 0.001
Denmark (*n* = 916)	72%	24%	Z = 13.96; *p* < 0.001
UK (*n* = 1,128)	58%	37%	Z = 7.40; *p* < 0.001

1 Z and *p*-values are from tests of proportional difference between insured cats and dogs within countries. Unweighted sample sizes are reported in the table.

**Table 6:** Percentage of households with insured dogs and cats in the three countries.

	Dogs	Cats	*P* value^1^
Austria (*n* = 984)	31%	12%	χ^2^(1) = 34.42; *p* < 0.001
Denmark (*n* = 703)	73%	27%	χ^2^(1) = 92.38; *p* < 0.001
UK (*n* = 831)	59%	40%	χ^2^(1) = 24.11; *p* < 0.001

1 Likelihood ratio χ^2^ test result from mixed effect logistic regression (0=no insurance; 1=insurance). Age, gender, income and reason for acquisition were included as control variables in the regression.

Unweighted sample sizes are reported in the table.

### 3.3. Willingness to pay for life-saving treatment

In Austria and Denmark, more dog than cat owners would be willing to spend a high amount on life-saving treatments (Table 7). This is also the case in the United Kingdom, but the difference is modest and not statistically significant. The proportion reported in the table further suggests that the relative difference in WTP is more pronounced in Denmark compared to the two other countries. Indeed, the difference is statistically significant when comparing Denmark and Austria [LR χ^2^(1) = 10.51, *p* < 0.01], and Denmark and the United Kingdom [LR χ^2^ (1) = 10.52, *p* < 0.01]. There is no statistically significant difference between Austria and the United Kingdom.

**Table 7:** Percentage of owners that are willing to pay high amount for life-saving treatment^1^ for cats and dogs – across countries.

	Dogs	Cats	*P* value^2^
Austria (*n* = 984)	41%	26%	χ^2^(1) = 17.81; *p* < 0.001
Denmark (*n* = 703)	27%	11%	χ^2^(1) = 23.11; *p* < 0.001
UK (*n* = 831)	34%	28%	χ^2^(1) = 3.12; *p* = n.s.

1 WTP high amount: Austria = 1,001€ or more (Austria); Denmark = 10.000 DKK or more; UK = £1,001 or more.

2 Likelihood ratio χ^2^ test result from mixed effect logistic regression (0=not high WTP; 1=high WTP). Age, gender, income and reason for acquisition were included as control variables in the regression.

Unweighted sample sizes are reported in the table.

### 3.4. Expectation of availability of veterinary treatment

In Table 8, we report the average number of veterinary treatment and diagnostic options expected by owners at their veterinary practices, where a higher number indicates expectation of a greater number of options. The results are divided into three kinds of household owner profile (only dog, only cat, dog and cat). This owner division is necessary, because the expectation questions were posed only once, even if respondents owned both a cat and a dog (see Table 1 for details about the different owner profiles employed in the analyses). The table also reports the proportion of owners that expected none/few (0–1), medium (2–4), and many (5–8) options. In all countries, expectation of veterinary equipment both in dog-only and dog/cat households was higher than in cat-only households, but to varying degrees. The difference in expectation between owner profiles in the United Kingdom was very modest, even though there was a statistically significant difference. Danish dog owners had higher expectations than Danish cat owners and owners that had both cats and dogs. In Austria, owners with cats had lower expectations than owners with dogs, or both dogs and cats, but the difference was not statistically significant in multivariable regression. The largest relative difference between households with dogs and cats was seen in Denmark (M_cats_ = 2.22 compared with M_dog_s = 3.22). Further tests confirmed that the difference in expectation across species owner profiles was higher in Denmark compared to Austria [LR χ^2^(2) = 8.34, *p* < 0.05]. However, we found no statistically significant difference on this point between Denmark and the United Kingdom and between Austria and the United Kingdom.

**Table 8:** Number of veterinary treatment and diagnostic options expected by each owner profile – across countries.^1^

Country	Owner profile	Mean	0–1 expected options	2–4 expected options	5–8 expected options	*P* value^2^
Austria	Dog (*n* = 225)	3.41	26%	44%	31%	LR χ^2^(2) =5.69; *p* = n.s.
	Cat (*n* = 391)	2.92	32%	42%	25%	
	Dog and cat (*n* = 184)	3.57	21%	45%	34%	
Denmark	Dog (*n* = 308)	3.22	33%	34%	33%	LR χ^2^(2) = 7.39; *p* < 0.05
	Cat (*n* = 241)	2.22	48%	32%	21%	
	Dog and cat (*n* = 77)	2.67	41%	35%	24%	
UK	Dog (*n* = 311)	3.72	29%	30%	41%	LR χ^2^(2) = 9.26; *p* = 0.01
	Cat (*n* = 240)	3.42	34%	28%	38%	
	Dog and cat (*n* = 140)	3.51	36%	29%	35%	

1 Row shares are reported regarding the expected options (grouped into 0–1, 2–4 and 5–8). Due to rounding error, the share may not sum to 100.

2 Likelihood ratio χ^2^ test results from ordered logistic regression. Age, gender, income and reason for acquisition were included as control variables in the regression.

Unweighted sample sizes are reported in the table.

## 4. Discussion

The results presented here indicate that while owners appear to care more about dogs than about cats in all three countries, the *degree* to which this is true *varied considerably*. Across all three countries studied, a higher LAPS score was identified for owners who chose a dog as their favorite pet rather than a cat. Further, significantly more dogs than cats were insured in all three countries. Similarly, when owners were asked to imagine that their dog or cat was suffering from a severe illness and would have to either undergo treatment (with a good chance of a successful outcome) or be euthanized, a higher percentage of dog owners than cat owners in all three countries were willing to spend more to save the animal’s life. However, when it comes to level of equipment expected at the veterinary practice/clinic owners usually attended with their pet, the picture is less clear; only Denmark had a clear dog vs. cat difference in owner expectation.

Further, in the United Kingdom, how much owners cared about dogs and cats did not differ very much, although there was a slight preference for dogs. In Austria, the difference was much stronger: dogs were clearly preferred; and in Denmark, the difference was stronger still. This speaks against the hypothesis that the prime explanation of cats’ lower rating in terms of human care simply concerns the difference between dog and cat behavior. This supports the idea that something more culturally specific is at work in terms of the relative degree to which people care about dogs and cats, in line with what we called the “culture hypothesis,” and contrasting with some other studies [notably (9)], with their focus on the role of cat behavior in (lack of) ability of humans to care about their cats.

Apart from ours, there have been a few other studies reporting results that differ from the general picture of owners always caring much more about dogs than about cats. Zasloff (6) found that the significant difference in attachment disappeared when dropping two questions closely linked to dog utility from the 13 questions asked (“My pet makes me feel safe” and “I get more exercise because of my pet”). This suggests that simple use values can contribute to differences in degree of care. González-Ramírez and Landero-Hernández (4) reported greater interaction and lower perceived costs with cats than with dogs (but still greater emotional closeness with dogs). The authors suggest that one reason for this finding is that cats in Mexico spend more time indoors than dogs.

The aforementioned findings from Mexico seem to be in line with our speculation about rural versus urban cats: the closer cats are to their owners by being indoors, the more people care about them. And the more cats are inside, the more their behavior may be owner-focused and dependent. This might suggest that if [as has been the case in the US (18)] norms evolve for a higher percentage of cats to be kept indoor-only, attachment levels will increase - including relative to dogs.

Relatedly, Nugent and Daugherty (3) found that dog owners were only slightly more bonded to their companion animal than cat owners. This finding is interesting for two reasons: (a) it is very recent; and (b) it comes from a country, the United States, where a large proportion of cats are indoor-only (19, 20). This too may support the idea that as cats become more proximate and move into human homes, the degree to which humans care about them increases.

However, while an intriguing assumption, the idea that care for cats increases in tandem with an increased share of indoor cats does not appear to explain the stark difference observed between Denmark and the United Kingdom in caring more about dogs relative to cats. Thus, both in the United Kingdom and Denmark, the majority of cats have some kind of outdoor access, while just one in four or five cats are kept strictly indoors (19), more specifically 17% in Denmark (21), and 26% in the United Kingdom (22).

Still, in light of our study and the three last mentioned studies there is some reason to think that when owners care less about cats is not a fact of nature, one that just flows from the animals’ perceived behavior. Instead it may vary depending on (or so we speculate) culturally shaped factors, including the degree of contact and dependence.

Our use of four measures (LAPS, the possession of health insurance, WTP, and expectation of availability of different levels of veterinary treatment) resulted in some additional cross-country differences that are noticeable. Particularly, there were fluctuating patterns in the general level of attachment and care identified in the three countries across the four measures. LAPS and WTP is lower in Denmark (for both dogs and cats). The expectation about veterinary diagnostic and treatment options is on most accounts also lower in Denmark. On the other hand, possession of pet health insurance is more common in Denmark (for both dogs and cats) compared to Austria. These fluctuations raise the question whether there are some measures that are more accurate representations of a caring relation than others. In light of our findings it seems reasonable to conclude that of the four measures besides LAPS, willingness to pay for life-saving treatments is a good measure of how much owners care about their companion animals.

We think that the finding that owners of both species of companion animals, but particularly cat owners, in Denmark have quite a low LAPS and willingness to pay for life-saving treatments compared to the two other countries, is also compatible with our suggestion for the explanation suggested earlier. Denmark is closer to its agricultural past, and in an agricultural setting, animals in general, including dogs, are typically kept more at arm’s length in terms of emotional attachment.

The findings of this study will be of relevance to the small animal veterinary sector, other commercial sectors, and NGOs with an interest in the future development of human relations to dogs and cats. Here, the main message is that the degree to which owners care about their dogs and cats is not limited or otherwise defined solely by the nature of the animals and may continue to evolve as human lifestyles change.

### 4.1. Strengths and limitations of the study

The main strengths of our study are that firstly, it makes use of four different measures of attachment and care and secondly, it compares representative samples of dog and cat owners across three countries.

However, there is room for discussion about the validity of two of the measures when it comes to comparing care for dogs and cats: Regarding insurance levels, veterinary treatment of dogs may be more expensive and therefore owners may be more prone to buy insurance for reasons that have nothing to do with how much they care about dogs vs. cats. Also treatment costs may vary between countries. A similar concern may be raised for the expectation of availability of veterinary treatment options, since there may be more such options available for dogs. While these queries may be a problem for our (and other studies’) measures, they also speak to our suggestion that the main explanation of different measured levels of attachment and care is not how dogs and cats typically behave toward their owners. Further the LAPS questionnaire items have only recently been translated into German and Danish, respectively, and thus the LAPS has only recently been studied in owners of companion animals in German speaking countries (1, 23) and in Denmark (21, 24). For this reason, the external validity of the scale in these two newly translated questionnaires is not clear. To our knowledge, there are also no studies dedicated to the assessment of the external validity of the LAPS for the English version of the questionnaire.

Another limitation of our study concerns differences in why and how people keep dogs and cats. We did compare and control for differences in the extent to which dogs and cats in the three countries were acquired for human companionship, and here we found very small differences. However, there may be other relevant differences regarding the role and status of the animals in their owners’ lives that we could not control for, for example their use in various hobbies and other activities. Also we did not control for the differences in the way members of the two species were housed.

It is, of course, also a limitation that our study only looks at three countries located in Central and Western Europe. Thus, it raises intriguing questions regarding what comparative studies of other countries might find. With such statistically significant differences between three relatively similar European countries, it is possible that very different results might emerge in south Asian or African countries (where, for instance, or high numbers of potentially dangerous stray dogs might negatively affect attitudes to dogs but not to cats). Perhaps there are countries where the level of care for and attachment to cats is, in fact, higher than dogs which is actually suggested by some literature (25).

To better understand the issues explored in this paper we recommend a more comprehensive study where more countries are included that vary in terms of regions of the world, degree of urbanization, and agricultural versus tertiary societal developments, and where within-household measures include indoor/outdoor access for cats.

## Data availability statement

The datasets presented in this study can be found in online repositories. The names of the repository/repositories and accession number(s) can be found at: https://doi.org/10.5281/zenodo.8286502.

## Ethics statement

The studies involving humans were approved by the Research Ethics Committee of SCIENCE and HEALTH at the University of Copenhagen (Ref: 504-010300/22-5000). The studies were conducted in accordance with the local legislation and institutional requirements. The participants provided their written informed consent to participate in this study.

## Author contributions

PS, SC, SS, and TL contributed to conception and design of the study. TL performed the statistical analysis and wrote sections of the manuscript. PS and CP wrote the first draft of the manuscript. All authors contributed to the article and approved the submitted version.

## Footnotes

^1^ https://doi.org/10.5281/zenodo.8286502
